# Systemic sclerosis and pregnancy outcomes: a retrospective study from a single center

**DOI:** 10.1186/s13075-022-02783-0

**Published:** 2022-04-27

**Authors:** Giuseppe Barilaro, Aleida Castellanos, Inês Gomez-Ferreira, Gema Maria Lledó, Carlo Della Rocca, Lorena Fernandez-Blanco, Ricard Cervera, Núria Baños, Francesc Figueras, Gerard Espinosa

**Affiliations:** 1 Department of Autoimmune Diseases, Hospital Clinic, Universitat de Barcelona, Villarroel, 08036 Barcelona, Catalonia Spain; 2 Department of Maternal-Fetal Medicine, BCNatal, Barcelona Center for Maternal-Fetal and Neonatal Medicine, Hospital Sant Joan de Déu and Hospital Clínic, Universitat de Barcelona, Barcelona, Catalonia Spain; 3 Department of Medico-Surgical Sciences and Biotechnologies, Sapienza University, 04100 Latina, Italy; 4 Institut d’Investigacions Biomèdiques August Pi i Sunyer (IDIBAPS), Barcelona, Spain

**Keywords:** Systemic sclerosis, Pregnancy, Scleroderma, Adverse pregnancy outcomes, Systemic lupus erythematosus, Antiphospholipid syndrome

## Abstract

**Background:**

Pregnancy in systemic sclerosis (SSc) patients is no more an infrequent event as it used to be, but literature data on pregnancy outcomes in women with SSc are scarce. The rate of preterm deliveries and intrauterine growth restriction (IUGR) seems to be increased, while the risk of miscarriages is controversial. Moreover, no study compared pregnancy outcomes in SSc with antiphospholipid syndrome (APS) and systemic lupus erythematosus (SLE). We performed a retrospective study to compare the pregnancy and disease outcomes of women with SSc with a cohort of age-matched women with systemic lupus erythematosus (SLE), antiphospholipid syndrome (APS), and healthy controls (HC).

**Methods:**

A total of 154 pregnancies from SSc, SLE, APS patients, and HC were prospectively followed at the High-Risk Pregnancy Unit of our center from 2008 to 2019. The primary outcome was a composite endpoint of miscarriages, fetal deaths, intrauterine growth restriction (IUGR), preeclampsia, neonatal deaths, preterm birth, and small-for-gestational-age (SGA) newborns. Single adverse pregnancy outcomes (APO) represented secondary endpoints. SSc activity variations in relation to pregnancy were assessed.

**Results:**

The risk of APO was significantly higher in SSc patients compared to HC (60.6% vs 10.0%; OR = 14.42; 95% CI 3.70–56.18, *p* = 0.001) and SLE patients (60.6% vs 37.5%; OR = 3.56; 95% CI 1.29–9.83, *p* = 0.014). Compared to HC, women with SSc had an increased frequency of first trimester miscarriage (15% vs 0 %; *p* = 0.016), preeclampsia (12% vs 0%, *p* = 0.038), and SGA newborns (21.2% vs 0%; *p* = 0.003). Preterm deliveries were more frequent in SSc pregnancies in comparison with HC (24.2% vs 5%; OR = 6.08; 95% CI 1.19–31.02, *p* = 0.036) and SLE patients (24.2% vs 7.5%, OR = 5.68; 95% CI 1.1–29.38, *p* = 0.038). Disease remained stable in all SSc patients during pregnancy and up to 1 year after delivery.

**Conclusions:**

We found an increased risk of APO in our SSc cohort in comparison with HC (with higher rates of miscarriages, preeclampsia, SGA newborns, and preterm deliveries) and SLE patients (presenting a higher rate of preterm deliveries). High-risk multidisciplinary management of SSc pregnant women is highly recommended.

## Background

Systemic sclerosis (SSc) is a complex and multifactorial autoimmune disease characterized by progressive fibrosis of the skin and visceral organs and non-inflammatory vasculopathy. The incidence in women is 4 to 9 times higher than that in men [1]. The mean age of onset of the disease is in the early 40s; therefore, most SSc patients are already mothers when the disease occurs. In recent years, the habit of delaying pregnancy because of social and economic reasons has become more common, increasing the possibility of getting pregnant after the onset of the disease. This fact makes pregnancy a more frequent issue to deal with in the present years.

The overall literature published so far, regarding pregnancies in SSc, reached divergent conclusions about pregnancy outcomes, while data about maternal diseases are scarce. Most of the studies published before 1990, which mainly consist of case series, showed bad maternal and fetal outcomes, with increased frequency of infertility, miscarriages, and maternal complications [2–8]. However, more recent literature [9–13], including one of the two prospective studies published to date [14], described far better results, with a rate of successful pregnancies similar to the general population and no increase in maternal mortality. For instance, in several studies [9, 10, 14], data about pregnancy outcomes were collected through questionnaires. Moreover, no study included in the control group patients with autoimmune diseases characterized by pregnancy morbidity [11–15].

In general, even though the overall obstetric result was good, there was no uniformity among the different studies, with discordant findings of increased risk of preterm births [10, 14, 16–18], intrauterine growth restriction (IUGR) [12, 18], small-for-gestational-age (SGA) newborns [9, 10, 18], or pre-eclampsia [12]. Interestingly, one study found that corticosteroids were associated to preterm deliveries, while folic acid intake and anti-topoisomerase antibodies were protective [18].

Regarding disease activity during pregnancy and puerperium, a substantial stability has been observed in most studies. However, several authors suggested that women with early (onset less than 4 years), diffuse cutaneous SSc (dcSSc), and positivity for anti-topoisomerase antibodies have a higher risk of disease progression [14, 18].

Antiphospholipid syndrome (APS), the most frequent acquired thrombophilia, and systemic lupus erythematosus (SLE) are frequently associated with pregnancy complications such as recurrent miscarriage, preeclampsia, placental insufficiency, and IUGR [19, 20].

The objective of the present study was to retrospectively assess the pregnancy and disease outcomes of women with SSc, whose pregnancies were prospectively followed in a standardized protocol at our high-risk pregnancy clinic, in comparison with those with SLE and APS and a healthy control (HC) group.

## Patients and methods

We identified 21 women with SSc who got pregnant after symptoms onset and whose pregnancies were followed at our high-risk pregnancy clinic between 2008 and 2019. As comparator groups, we selected 26 pregnant women with SLE and 31 with primary APS attending our high-risk pregnancy clinic in the same period. In addition, a group of 40 healthy women, whose pregnancies were followed at our center between 2010 and 2019, was also included. Patients with SSc, those with APS and SLE, and healthy women were age-matched.

All patients were prospectively followed up during the whole pregnancy and up to 1 year after delivery. The following variables were collected at each follow-up visit during pregnancy in SSc patients: heart rate, blood pressure, and signs or symptoms suggestive of organ or system involvement related to SSc such as Raynaud’s phenomenon, digital ulcers, and any other skin, articular, pulmonary, cardiac, renal, or gastrointestinal manifestation. Lung, heart, and kidney involvements were assessed from the clinical and laboratory point of view according to several authors [21, 22]. In case of high clinical suspicion of clinical manifestations related to SSc, specific tests such as pulmonary function test or Doppler echocardiogram were performed. In addition, fetal growth rates and fetoplacental Doppler flow kinetics were evaluated by serial ultrasonographic examination from the first trimester onward.

The primary outcome of the study was a composite endpoint of adverse pregnancy outcomes (APO) defined as the following events: (a) miscarriages: pregnancy loss < 10 weeks of gestation; (b) fetal death > 10 weeks of gestation; (c) neonatal death before hospital discharge due to complications of prematurity and/or placental insufficiency; (d) preeclampsia: new onset of hypertension (systolic blood pressure [BP] ≥ 140 mmHg or diastolic BP ≥ 90 mmHg) and proteinuria (> 300 mg/24 h) or end-organ dysfunction or both after 20 weeks of gestation or during puerperium; (e) preterm birth: delivery < 37 weeks due to placental insufficiency, gestational hypertension, or preeclampsia; (f) IUGR assessed by ultrasound as a fetal abdominal circumference below the 5th percentile; (g) SGA newborn: birth weight < percentile 5 for gestational age, in the absence of anatomical malformations or genetic alterations [23].

The study was conducted in accordance with the Declaration of Helsinki. Since it was a retrospective study, the Hospital Clínic Ethics Committee waived the requirements for approval. All patients and controls gave their informed consent to participate and publish the study results.

### Statistical analysis

Categorical variables were compared using the chi-square test or Fisher’s exact test whenever appropriate, while Student’s 2-tailed *T*-test was used for continuous variables. A two-tailed *p* value < 0.05 was considered statistically significant. Logistic regression models were used for multivariate analyses to adjust for the effect of possible confounders. The strength of each association was expressed as odds ratios (OR) with 95% confidence interval (CI). Data were analyzed using the SPSS 25.0 package (SPSS Inc., Chicago, IL, USA).

## Results

### General characteristics

The study included 21 SSc patients with a total of 33 pregnancies. All pregnancies were spontaneous, except 4 that were achieved through assisted reproductive technology. For instance, one patient had 5 pregnancies, 8 patients had 2 pregnancies, and the remaining had one pregnancy. There were three twin pregnancies.

Sixteen (76%) among the 21 patients fulfilled the 2013 revised American College of Rheumatology/European League Against Rheumatism (ACR/EULAR) criteria [24] while 5 fulfilled the 2001 LeRoy and Mesdger criteria for early SSc [25]. Among the five patients with early SSc, one patient presented Raynaud’s phenomenon, anti-Scl70 antibody positivity, and esophagoeal dysmotility documented by manometry; one patient classified as sine scleroderma SSc had Raynaud’s, puffy fingers, and a typical capillaroscopic pattern with hemorrhages and dilated capillaries; one had Raynaud’s, esophageal involvement, and anticentromere antibody positivity; and the last two had Raynaud’s, anti-centromere positivity, telangiectasias, and a not specific although abnormal capillaroscopy.

Nine (42.9%) patients had limited cutaneous SSc (lcSSc), two (9.5%) had dcSSc, and ten (47.6%) had sine-scleroderma (ssSSc). The main demographic characteristics, cumulative clinical manifestations, immunological features, and treatment of SSc patients are shown in Table 1.

**Table 1:** Demographic characteristics, clinical manifestations, immunological features, and treatment of patients with SSc

Disease subtype (*n* of patients)	Diffuse SSc (*n* = 2)	Limited SSc (*n* = 9)	Sine SSc (*n* = 10)
Maternal age at delivery (y)	38.8 ± 1.15	34.3 ± 4.69	36.0 ± 3.23
Disease duration at conception (y)	12 ± 10.4	6.4 ± 5.2	4.45 ± 5.7
Cumulative clinical manifestations			
Raynaud’s phenomenon	2 (100)	9 (100)	10 (100)
Digital ulcers	2 (100)	3 (33.3)	1 (10)
Arthritis	1 (50)	2 (22.2)	0
Telangiectasia	2 (100)	4 (44.4)	0
Calcinosis	1 (50)	2 (22.2)	0
Puffy fingers/sclerodactyly	2 (100)	6 (66.7)	0
Esophageal dysmotility	2 (100)	4 (44.4)	3 (30)
Interstitial lung disease	2 (100)	4 (44.4)	1 (10)
FVC (%)	80 ± 21.7	84.6 ± 16.9	99.3 ± 8.4
DLCO (%)	68.3 ± 21.3	69.6 ± 22	78.3 ± 11.9
Pulmonary arterial hypertension	0	0	0
Scleroderma renal crisis	0	0	0
Immunological features			
Antinuclear antibodies	2 (100)	9 (100)	10 (100)
Anti-Scl70 antibody	1 (50)	2 (22.2)	1 (10)
Anti-centromere antibody	0	2 (22.2)	6 (60)
Anti-RNA polymerase antibody	1 (50)	2 (22.2)	1 (10)
Anti-cardiolipin antibodies	0	0	0
Anti-β2GPI antibodies	0	1 (11.1)	0
Lupus anticoagulant	0	0	0
Anti-Ro/SSA antibody	0	3 (33.3)	2 (0)
Treatment at conception			
Calcium channel blockers	0	4 (44.4)	3 (33.3)
ERA	0	1 (11.1)	1 (10)
PDA-5 inhibitors	0	0	0
Proton pump inhibitors	1 (50)	1 (11.1)	2 (20)
Corticosteroids	1 (50)	3 (33.3)	1 (10)

Categorical variables are expressed as number (percentage) and continuous variables as mean ± standard deviation

*Abbreviations*: *SSc* systemic sclerosis, *y* years, *FVC* forced vital capacity, *DLCO* diffuse lung capacity of carbon monoxide, *β2GPI* beta2-glicoprotein I, *ERA* endothelin receptor antagonist, *PDA-5* phosphodiesterase type 5

Overall, we included 121 control pregnancies, 40 from 26 SLE patients, 41 from 31 APS patients, and 40 healthy controls. The mean disease duration at conception in SSc patients was 6.0 (SD 5.43) years, being 9.4 (SD 5.98) and 2.5 (SD 4.43) years for SLE and APS patients, respectively.

### Adverse pregnancy outcomes

Pregnancy outcomes in the entire series are reported in Table 2 while Table 3 reports the pregnancy outcomes for the different SSc subsets. Adjusted OR of the main outcomes are shown in Table 4*.* In the multivariate analysis including confounders smoking, hypertension, and corticosteroid intake, the overall risk of APO resulted significantly higher in SSc patients compared to HC (60.6% vs 10.0%; OR = 14.42; 95% CI 3.70–56.18, *p* = 0.001) and SLE patients (60.6% vs 37.5%; OR = 3.56; 95% CI 1.29–9.83, *p* = 0.014) while, in comparison with APS patients, it did not reach the statistical significance (60.6% vs 36.6%; OR = 2.76; 95% CI 0.95–8.07, *p* = 0.06). After excluding the three twin pregnancies, the risk of APO was still increased in SSc patients in comparison with HC (56.7% vs 10.0%; OR = 11.77; 95% CI 3.34–41.51, *p* = 0.001) while not reaching statistical significance when compared to SLE patients (56.7% vs 37.5%; OR = 2.18; 95% CI 0.83–5.72, *p* = 0.11).

**Table 2:** Pregnancy outcomes of SSc, APS, SLE patients, and healthy controls

	SSc (*n* = 21)	SLE (*n* = 26)	APS (*n* = 31)	HC (*n* = 40)
Total no. of pregnancies	33	40	41	40
Maternal age at delivery (years, mean ± SD)	35.4 ± 4.1	34.6 ± 5.1	35.3 ± 3.7	33.3 ± 5.6
Disease duration at conception (years, mean ± SD)	6.0 ± 5.4	9.4 ± 5.9	2.5 ± 4.4	NA
Gestational age at delivery (weeks, mean ± SD)	31.6 ± 11.7	36.9 ± 8.6	34.1 ± 9.4	39.1 ± 1.8
Adverse pregnancy outcome	20 (60.6)	15 (37.5)^a^	15 (36.6)^b^	4 (10)^c^
Total number of fetal losses	6 (18.18)	4 (10)	6 (16.6)	0
1st trimester (miscarriage)	5 (15.2)	4 (10)	3 (7.3)	0^d^
2nd trimester	0	0	2 (4.9)	0
3rd trimester	1 (3)	0	1 (2.4)	0
Voluntary and therapeutic abortions	1 (3)	0	0	0
Neonatal deaths	0	0	4 (9.8)	0
IUGR (< 5th percentile)	5 (15.2)	5 (12.5)	5 (12.2)	2 (5)^e^
Small for gestational age newborns	7 (21.2)	2 (5.0)	8 (19.5)	0 (0)^f^
Preterm delivery (< 37 weeks of gestation)	8 (24.2)	3 (7.5)^g^	9 (22)	2 (5)^h^
Preeclampsia	4 (12.12)	4 (10)	4 (9.8)	0 (0)^i^
Eclampsia	0	0	0	0
Cesarean delivery	6 (18.2)	9 (22.5)	14 (34.1)	8 (20)
Elective	4 (12.1)	3 (7.5)	5 (12.2)	8 (20)
Non-elective	2 (6.1)	6 (15)	9 (21.9)	0 (0)

Values are expressed as numbers (%) of total pregnancies

*Abbreviations*: *SSc* systemic sclerosis, *SLE* systemic lupus erythematosus, *APS* antiphospholipid syndrome, *HC* healthy controls, *IUGR* intrauterine growth restriction

^a^*p* = 0.014 SSc compared to SLE

^b^*p* = 0.06 SSc compared to APS

^c^*p* = 0.001 SSc compared to HC

^d^*p* = 0.016 SSc compared to HC

^e^*p* = 0.143 SSc compared to HC

^f^*p* = 0.003 SSc compared to HC

^g^*p* = 0.038 SSc compared to SLE

^h^*p* = 0.036 SSc compared to HC

^i^*p* = 0.038 SSc compared to HC

**Table 3:** Pregnancy outcomes of different systemic sclerosis subsets

	dcSSc (*n* = 2)	lcSSC (*n* = 9)	ssSSc (*n* = 10)
Total no. of pregnancies	3	17	13
Maternal age at delivery (years, mean ± SD)	38.8 ± 1.15	34.3 ± 4.7	35.1 ± 2.6
Disease duration at conception (years, mean ± SD)	12.0 ± 10.4	6.4 ± 5.2	4.5 ± 5.7
Gestational age at delivery (weeks, mean ± SD)	35.5 ± 0.5	33.6 ± 9.3	31.8 ± 12.9
Adverse pregnancy outcome	3 (100)	9 (52.9)	8 (61.5)
Total number of fetal losses	0 (0)	2 (11.8)	4 (30.8)
1st trimester (miscarriage)	0 (0)	1 (5.9)	4 (30.8)
2nd trimester	0 (0)	0 (0)	0 (0)
3rd trimester	0 (0)	1 (5.9)	0 (0)
Voluntary and therapeutic abortions	0 (0)	1 (5.9)	0 (0)
Neonatal deaths	0 (0)	0 (0)	0 (0)
IUGR (< 5th percentile)	1 (33.3)	2 (11.8)	2 (15.4)
Small for gestational age newborns	1 (33.3)	4 (23.5)	2 (15.4)
Preterm delivery (< 37 weeks of gestation)	3 (100) ^a^	3 (17.6)	2 (15.4)
Preeclampsia	1 (33.3)	1 (5.9)	2 (15.4)
Eclampsia	0	0	0
Cesarean delivery	1 (33.3)	4 (23.5)	1 (10)
Elective	0 (0)	3 (17.6)	1 (10)
Non-elective	1 (33.3)	1 (5.9)	0 (0)

Values are expressed as numbers (%) of total pregnancies

*Abbreviations*: *dcSSc* diffuse cutaneous systemic sclerosis, *lcSSc* limited cutaneous systemic sclerosis, *ssSSc* systemic sclerosis sine scleroderma

^a^*p* = 0.003 dcSSc compared to lcSSc and ssSSc

**Table 4:** Adjusted odds ratios and 95% CI of the main outcomes: comparison between SSc, APS, SLE patients, and HC

	SSc vs HC	SSc vs SLE	SSc vs APS
Adverse pregnancy outcomes	OR = 14.42 (95% CI 3.70–56.18)	OR = 3.56 (95% CI 1.29–9.83)	NS
Preterm delivery	OR = 6.74 (95% CI 1.29–35.09)	OR = 5.68 (95% CI 1.1–29.37)	NS

OR of main outcomes adjusted for smoking, hypertension, and corticosteroids intake

*Abbreviations*: *SSc* systemic sclerosis, *HC* healthy controls, *SLE* systemic lupus erythematosus, *APS* antiphospholipid syndrome

Overall, there were 6 (21.2%) spontaneous pregnancy losses in the SSc group, 5 of them during the first trimester and one in the third trimester. Regarding the first trimester miscarriages, there were two peri-implantation, two embryonic, and one pre-embryonic miscarriage. For instance, four out of 5 miscarriages occurred in two patients with ssSSc, one fulfilling the ACR/EULAR criteria and the other classified as early SSc; the remaining miscarriage and the third trimester loss occurred in two patients with lcSSc, while none of the three dcSSc pregnancies ended in pregnancy loss. No miscarriage was associated with the presence of antiphospholipid antibodies. First trimester miscarriages were more frequent in SSc patients in comparison with healthy controls (15.2 vs 0 %, *p* = 0.016) but not SLE and APS.

Preeclampsia was diagnosed in four (12.1%) of the 33 SSc pregnancies, being severe in one case. For instance, three among four were twin pregnancies. The frequency was higher compared to healthy controls (12.1% vs 0%, *p* = 0.038), while not being increased in comparison with SLE and APS patients. IUGR was detected in five SSc pregnancies, being more frequent when compared to healthy controls, although not reaching statistical significance (15.2% vs 5%, *p* = 0.143), but not to APS and SLE patients. SGA newborns were significantly more frequent in SSc patients in comparison with HC (21.2% vs 0%; *p* = 0.003), while not with SLE (21.2% vs 5.0%, *p* = 0.069) and APS (21.2% vs 19.5%, *p* = 0.857).

There were 8 cases of preterm birth in our SSc population, one of them occurring in a patient with early SSc. Preterm deliveries were more frequent in SSc pregnancies compared to HC (24.2% vs 5%; OR = 6.08; 95% CI 1.19–31.02, *p* = 0.036) and SLE patients (24.2% vs 7.5%, OR = 5.68; 95% CI 1.1–29.38, *p* = 0.038) but not to APS (24.2% vs 22%, *p* = 0.816). The association was confirmed after adjusting for arterial hypertension and smoking as confounding variables. Interestingly, all the three patients with dcSSc had preterm deliveries, with a statistically significant association in comparison with other SSc subsets (*p* = 0.03). SSc patients who experienced preterm deliveries had a higher frequency of IUGR (25% vs 12.0% in patients without prematurity), SGA (37.5% vs 16%), and corticosteroids use (50% vs 20%), but in all cases, the association did not reach the statistical significance (*p* = 0.57, *p* = 0.32, and *p* = 0.12, respectively). Neither disease duration at conception nor antibody pattern correlated with preterm deliveries.

There was no difference in the frequency of cesarean sections in our SSc cohort in comparison with the control groups. In total, there were 4 cases of elective cesarean delivery: two because of IUGR and the remaining because of twin pregnancy and hip prosthesis each. Two cases of non-elective cesarean delivery occurred, one because of fetal dystocia and the second because of severe preeclampsia.

No cases of neonatal or perinatal death were documented, and no newborn had immediate complications or had to be admitted to the intensive care unit. One infant was born with a major congenital malformation, a Holt-Oram syndrome detected post-delivery.

### Effects of pregnancy on SSc

SSc remained stable in all patients, and there were no cases of disease progression during the whole pregnancy and up to 1 year after delivery. No cases of scleroderma renal crisis were observed. One patient with twin pregnancy and severe preeclampsia complicated by hemolysis, elevated liver enzymes, and a low platelet count (HELLP) syndrome, who led to elective cesarean, presented in immediate puerperium a severe hypertension with pulmonary edema and acute respiratory failure. The patient was intubated and treated with aggressive anti-hypertensive and diuretic treatment with full recovery.

## Discussion

SSc is a rare disease, and pregnancy after disease onset used to be anecdotal until recent years. To our knowledge, this is the first study reporting that SSc patients have a higher risk of adverse pregnancy outcomes (a composite endpoint including fetal losses, IUGR, SGA newborns, preeclampsia, preterm births, and neonatal deaths) when compared to SLE patients and HC, while having a considerable risk, even though not significantly higher, in comparison with APS. Therefore, a strict monitoring along with a multidisciplinary management for these patients is warranted, and these pregnancies should be considered as high-risk ones.

A recent systematic review and meta-analysis [26] including articles published between 1950 and 2018 reported an increased rate of miscarriages, IUGR, preterm births, newborns with low birth weight, gestational hypertension, and cesarean delivery. Our results confirm most of these findings. In fact, in our series, we found an increased risk of first trimester miscarriages, IUGR, SGA newborns, preeclampsia, and preterm births.

For instance, miscarriages were more frequent in our SSc population in comparison with healthy controls. The association between SSc and miscarriages confirms the results of the meta-analysis by Blagojevic et al. [26]. However, four of the 5 miscarriages detected in our population occurred in 2 patients, and the possibility of other factors influencing the outcome cannot be excluded. Moreover, it must be considered that there were no miscarriages in our HC group, which is lower than expected.

SGA newborns rates were significantly higher in SSc patients in comparison with HC. Chakravarty et al. [12] and Taraborelli et al. [18] found a higher frequency of IUGR and very-low-birth-weight newborns in their cohorts. A possible explanation for this phenomenon may be found in placental vasculopathy, as shown by pathological findings of the placenta from SSc patients, which included decidual vasculopathy with stromal fibrosis, placental mesenchymal villous dysplasia, infarcts, and reduction of uteroplacental perfusion [27].

The frequency of preeclampsia was higher in our SSc population compared to healthy controls, as found by several authors [12, 16]. However, three among 4 cases of preeclampsia were documented in twin pregnancies, where preeclampsia risk is higher. Therefore, this association might be incidental, and further data are needed.

Preterm delivery rate was high in SSc patients, being higher than in HC and SLE patients. Prematurity was more frequent in diffuse SSc than other subsets, as found by other authors [14, 26]. In the Italian Multicentric Study on Pregnancy in Systemic Sclerosis (IMPRESS) study, the authors found that patients who experienced preterm deliveries had a higher rate of IUGR and corticosteroid use, while folic acid intake and anti-topoisomerase antibodies were protective [18]. These results were not confirmed in our cohort, even though the frequency of IUGR and corticosteroids intake seemed to be higher in SSc patients who experienced prematurity. This might be due to a lack of power because of our relatively small sample. Finally, the protective effect of anti-topoisomerase antibodies was not confirmed.

In concordance with most recent literature [18, 26], the disease remained stable, and no increased activity, clinical worsening, or scleroderma renal crisis has been registered during the whole pregnancy and up to 1 year after delivery. The fact that our population did not include patients with a disease duration of less than 4 years might have favored this outcome. Conversely, no improvement in several disease manifestations, such as Raynaud’s phenomenon or digital ulcers, has been registered. However, all our SSc patients have been for a quite a long time in a stable phase of the disease when they got pregnant; therefore, no improvement was expected.

The main strength of our study is the fact that all pregnancies were prospectively followed in our autoimmune-obstetric clinic, so data about pregnancy course were directly collected and not assessed via questionnaires as in several previous studies or, as in one case, through an administrative database [12]. Moreover, most patients fulfilled the SSc ACR/EULAR criteria guaranteeing uniformity of our population [24]. The control groups were followed prospectively as well, which makes the main difference compared to all former studies. For instance, the reported prevalence of each obstetric manifestation in all the study groups (including healthy controls) was real and not drawn from the literature. Furthermore, comparing SSc pregnancies to other autoimmune diseases implicated in pregnancy morbidity, such as SLE and APS, has provided novel information, as the increased rate of APO in SSc patients compared to SLE and the higher frequency of preterm delivery in SSc compared to SLE patients.

Our study also has several limitations: first, its retrospective design. Second, the small sample due to its unicentric design, which makes it possible that a type II error occurred when analyzing the association with the various outcomes. Moreover, the fact that not all patients were tested for the whole spectrum of SSc-specific antibodies may limit the range of our results. Finally, we have to point out that there were no miscarriages in our HC group. Early pregnancy loss is a quite common APO, with a prevalence of 10–15% in the general population [28]. Perhaps, since our healthy controls were also followed in our high-risk clinic, this might be the reason for such a good outcome. Besides, we could not assess the presence of chromosomal abnormalities in SSc patients who suffered from early miscarriages. Therefore, even though we found a higher frequency of miscarriages in SSc compared to HC, this aspect needs to be further investigated.

## Conclusions

In summary, our results indicate that the risk of overall APO, for instance, miscarriages, SGA newborns, preeclampsia, and premature deliveries, is increased in SSc pregnant women. Pregnant women with SSc should be considered at increased risk of adverse outcomes. It is mandatory to perform a pre-pregnancy counseling and refer them to specialized high-risk pregnancy units where they can be monitored very closely by a multidisciplinary team.

The frequency of pregnancies in SSc patients has notably increased in recent years, and women and their health care providers need extensive information about the possible outcomes of their pregnancies. Further studies, likely multicentric, with larger patient populations, are needed to improve our knowledge [29]. A large, multicenter, prospective observational study of pregnancy in SSc (International Multicentric Study on PREgnancy in Systemic Sclerosis (IMPRESS 2)) is currently ongoing and results are awaited.

## Data Availability

The dataset supporting the conclusions of this article is available from the corresponding author upon reasonable request.

## Abbreviations

SSc: Systemic sclerosis
IUGR: Intrauterine growth restriction
SGA: Small-for-gestational-age
dcSSC: Diffuse cutaneous systemic sclerosis
SLE: Systemic lupus erythematosus
APS: Antiphospholipid syndrome
HC: Healthy controls
HELLP: Hemolysis, elevated liver enzymes, and a low platelet count

## References

[1] Simeon-Aznar CP, Fonollosa-Pla V, Tolosa-Vilella C, Espinosa-Garriga G, Ramos-Casals M, Campillo-Grau M, Garcia-Hernandez FJ, Castillo-Palma MJ, Sanchez-Roman J, Callejas-Rubio JL (2012). Registry of the Spanish network for systemic sclerosis: clinical pattern according to cutaneous subsets and immunological status. Semin Arthritis Rheum.

[2] Giordano M, Valentini G, Lupoli S, Giordano A (1985). Pregnancy and systemic sclerosis. Arthritis Rheum.

[3] Ballou SP, Morley JJ, Kushner I (1984). Pregnancy and systemic sclerosis. Arthritis Rheum.

[4] Black CM, Stevens WM (1989). Scleroderma. Rheum Dis Clin North Am.

[5] Winkelman E (1962). Pregnancy in advanced systemic sclerosis (scleroderma). J Am Med Womens Assoc.

[6] Scarpinato L, Mackenzie AH (1985). Pregnancy and progressive systemic sclerosis. Case report and review of the literature. Cleve Clin Q.

[7] Slate WG, Graham AR (1968). Scleroderma and pregnancy. Am J Obstet Gynecol.

[8] Silman AJ, Black C (1988). Increased incidence of spontaneous abortion and infertility in women with scleroderma before disease onset: a controlled study. Ann Rheum Dis.

[9] Steen VD, Conte C, Day N, Ramsey-Goldman R, Medsger TA (1989). Pregnancy in women with systemic sclerosis. Arthritis Rheum.

[10] Steen VD, Medsger TA (1999). Fertility and pregnancy outcome in women with systemic sclerosis. Arthritis Rheum.

[11] Sampaio-Barros PD, Samara AM, Marques Neto JF (2000). Gynaecologic history in systemic sclerosis. Clin Rheumatol.

[12] Chakravarty EF, Khanna D, Chung L (2008). Pregnancy outcomes in systemic sclerosis, primary pulmonary hypertension, and sickle cell disease. Obstet Gynecol.

[13] Bernatsky S, Hudson M, Pope J, Vinet E, Markland J, Robinson D, Jones N, Docherty P, Abu-Hakima M, Leclercq S (2008). Assessment of reproductive history in systemic sclerosis. Arthritis Rheum.

[14] Steen VD (1999). Pregnancy in women with systemic sclerosis. Obstet Gynecol.

[15] van Wyk L, van der Marel J, Schuerwegh AJ, Schouffoer AA, Voskuyl AE, Huizinga TW, Bianchi DW, Scherjon SA (2011). Increased incidence of pregnancy complications in women who later develop scleroderma: a case control study. Arthritis Res Ther.

[16] Chen JS, Roberts CL, Simpson JM, March LM (2015). Pregnancy outcomes in women with rare autoimmune diseases. Arthritis Rheumatol.

[17] Chung L, Flyckt RL, Colon I, Shah AA, Druzin M, Chakravarty EF (2006). Outcome of pregnancies complicated by systemic sclerosis and mixed connective tissue disease. Lupus.

[18] Taraborelli M, Ramoni V, Brucato A, Airo P, Bajocchi G, Bellisai F, Biasi D, Blagojevic J, Canti V, Caporali R (2012). Brief report: successful pregnancies but a higher risk of preterm births in patients with systemic sclerosis: an Italian multicenter study. Arthritis Rheum.

[19] Pierangeli SS, Leader B, Barilaro G, Willis R, Branch DW (2011). Acquired and inherited thrombophilia disorders in pregnancy. Obstet Gynecol Clin North Am.

[20] Lee LA (2004). Neonatal lupus: clinical features and management. Paediatr Drugs.

[21] Sobanski V, Launay D, Depret S, Ducloy-Bouthors AS, Hachulla E (2016). Special considerations in pregnant systemic sclerosis patients. Expert Rev Clin Immunol.

[22] Giles I, Yee CS, Gordon C (2019). Stratifying management of rheumatic disease for pregnancy and breastfeeding. Nat Rev Rheumatol.

[23] Buyon JP, Kim MY, Guerra MM, Laskin CA, Petri M, Lockshin MD, Sammaritano L, Branch DW, Porter TF, Sawitzke A (2015). Predictors of pregnancy outcomes in patients with lupus: a cohort study. Ann Intern Med.

[24] van den Hoogen F, Khanna D, Fransen J, Johnson SR, Baron M, Tyndall A, Matucci-Cerinic M, Naden RP, Medsger TA, Carreira PE (2013). 2013 classification criteria for systemic sclerosis: an American College of Rheumatology/European League against Rheumatism collaborative initiative. Arthritis Rheum.

[25] LeRoy EC, Medsger TA (2001). Criteria for the classification of early systemic sclerosis. J Rheumatol.

[26] Blagojevic J, AlOdhaibi KA, Aly AM, Bellando-Randone S, Lepri G, Bruni C, Moggi-Pignone A, Guiducci S, Mecacci F, Matucci-Cerinic M (2020). Pregnancy in systemic sclerosis: results of a systematic review and metaanalysis. J Rheumatol.

[27] Ibba-Manneschi L, Manetti M, Milia AF, Miniati I, Benelli G, Guiducci S, Mecacci F, Mello G, Di Lollo S, Matucci-Cerinic M (2010). Severe fibrotic changes and altered expression of angiogenic factors in maternal scleroderma: placental findings. Ann Rheum Dis.

[28] Quenby S, Gallos ID, Dhillon-Smith RK, Podesek M, Stephenson MD, Fisher J, et al. Miscarriage matters: the epidemiological, physical, psychological, and economic costs of early pregnancy loss. Lancet. 2021;397(10285):1658–67. 10.1016/S0140-6736(21)00682-6 33915094

[29] Barilaro G, della Rocca C, Espinosa G. Obstetric outcomes in systemic sclerosis: looking inside the sealed box. Rheumatol Int. 2022;42(5):921–2. 10.1007/s00296-021-04982-5 34471973

